# Effectiveness of Telerehabilitation‐Delivered LSVT‐BIG on Motor Function in Chronic Stroke Patients: A Single‐Subject Experimental Study

**DOI:** 10.1155/oti/2901762

**Published:** 2026-02-27

**Authors:** Seon-A Jeong, Deok-Gi Hong, In-Tae Choi

**Affiliations:** ^1^ Department of Occupational Therapy, The Graduate School of Wonkwang University, Iksan, Republic of Korea; ^2^ Department of Occupational Therapy, College of Medicine, Wonkwang University, Iksan, Republic of Korea, wku.ac.kr; ^3^ Department of Occupational Therapy, Presbyterian Medical Center, Jeonju, Republic of Korea

**Keywords:** activities of daily living, LSVT-BIG, occupational performance, physical function, stroke, telerehabilitation

## Abstract

**Objective:**

This study is aimed at determining the effects of telerehabilitation‐delivered LSVT‐BIG on physical function, occupational performance, and activities of daily living in patients with stroke.

**Method:**

The participants included three patients who had been diagnosed with stroke for > 6 months. This study used the ABA single‐subject study design. The study process was conducted for a total of 24 sessions over 6 weeks, including four sessions of baseline (A), 16 sessions of intervention using ZOOM (B), and four sessions of re‐baseline (A ^′^). During the intervention period (B), the intervention was conducted four times a week for 4 weeks for 60 min each time, according to the standardized LSVT‐BIG protocol. Repeated measures assessments included the Timed Up and Go (TUG) and the Box and Block Test (BBT) at each session. The Canadian Occupational Performance Measure (COPM), modified Barthel index (MBI), and Fugl‐Meyer assessment (FMA) were used to assess changes before and after the intervention. Descriptive statistics and visual analyses were used for data analysis.

**Results:**

After the telerehabilitation‐delivered LSVT‐BIG intervention, all participants showed improvement in physical function. The TUG performance time decreased in each session, and the gait and balance improved. Both the BBT and FMA‐affected side scores of both upper limbs increased compared with those before the intervention, indicating improvement in upper limb function. In terms of occupational performance and activities of daily living, the COPM performance and satisfaction scores and the MBI scores both increased after the intervention.

**Conclusion:**

The positive clinical applicability of telerehabilitation‐delivered LSVT‐BIG intervention for patients with stroke was confirmed. In the future, follow‐up studies on LSVT‐BIG intervention that expand the benefits of telerehabilitation are needed.

## 1. Introduction

Stroke is one of the leading causes of death and disability worldwide and remains the most common cerebrovascular disease [1]. More than 60% of individuals with stroke experience neurological impairments, including hemiplegia, sensory and perceptual deficits, and cognitive impairments [2]. Approximately 25% of individuals with stroke continue to experience gait impairments, and nearly 50% remain limited in performing activities of daily living [3]. Rehabilitation reduces poststroke disability and supports independent living [4], and repetitive rehabilitation training is essential promoting neuroplasticity [5].

In the field of stroke rehabilitation, neuroplasticity‐based interventions such as action observation training, mirror therapy, robot‐assisted training, and virtual reality training have been increasingly applied [6]. However, current rehabilitation practices tend to focus primarily on the recovery of physical function, with limited consideration of occupational performance [7]. To address this limitation, Lee Silverman Voice Treatment‐BIG (LSVT‐BIG) has been proposed as an occupational performance oriented rehabilitation approach. Originally developed for individuals with Parkinson′s disease, LSVT‐BIG is a motor‐ and occupational performance‐based intervention grounded in principles of neural plasticity, emphasizing the recalibration of movement amplitude through intensive, repetitive, and salient task‐oriented training [8, 9]. The application of the LSVT‐BIG intervention has been shown to improve motor function, occupational performance, and quality of life in patients with Parkinson′s disease [9, 10]. Recently, studies have reported the effects of the LSVT‐BIG intervention in individuals with stroke [11–14]. Previous studies have demonstrated that LSVT‐BIG improves motor function, occupational performance, and quality of life in individuals with Parkinson′s disease [9, 10]. More recently, emerging evidence has supported the application of LSVT‐BIG in individuals with stroke, reporting improvements in upper extremity function, balance, gait, occupational performance, and quality of life [11–14]. Overall, these findings suggest that LSVT‐BIG may serve as a comprehensive rehabilitation approach for enhancing functional outcomes in individuals with stroke.

Patients with stroke often face challenges in receiving continuous rehabilitation after discharge due to financial burdens, prolonged hospitalization, and limited access to medical facilities and rehabilitation specialists [15, 16]. To address these barriers, telerehabilitation has gained attention as an alternative approach, as it enables remote assessment, treatment, and monitoring through communication technologies while offering advantages in terms of time, distance, and cost efficiency [17–19]. Moreover, studies comparing telerehabilitation with face‐to‐face rehabilitation reported no significant differences between the two approaches [18]. Therefore, telerehabilitation therapy may be attempted as a new approach to stroke rehabilitation. Although telerehabilitation has been used to deliver LSVT‐BIG, existing studies have focused primarily on individuals with Parkinson′s disease. These studies reported improvements in gait, balance, and quality of life through video‐based or real‐time remote LSVT‐BIG interventions and demonstrated outcomes comparable with therapist‐delivered sessions [20–23]. However, despite these promising findings, research applying telerehabilitation‐delivered LSVT‐BIG to individuals with stroke remains lacking, underscoring the need to investigate its feasibility and potential benefits in this population.

Therefore, this study is aimed at investigating the effects of telerehabilitation‐delivered LSVT‐BIG on physical function, occupational performance, and activities of daily living in patients with stroke based on previous studies that confirmed the clinical applicability of LSVT‐BIG intervention in patients with stroke.

## 2. Method

### 2.1. Participants

This study included three individuals diagnosed with stroke who were hospitalized at a general hospital in Jeollabuk‐do, South Korea. The inclusion criteria are as follows: (1) those diagnosed with stroke and with a disease duration of 6 months or longer; (2) those who can walk independently (including using assistive devices); (3) those who can communicate with a score of 24 or higher on the Korean version of the Mini‐Mental State Examination (MMSE‐K). The MMSE‐K was selected as the cognitive screening tool based on previous research that applied LSVT‐BIG in individuals with stroke [13]. An unauthorized version of the Korean MMSE was used by the study team without permission; however, this has now been rectified with PAR. Permission to use the MMSE in this study was obtained from PAR.; (4) those who have a Brunnstrom recovery stage of three or higher in the proximal and distal parts of the affected upper limb; and (5) those without cardiopulmonary disease who can perform aerobic exercise. The study was conducted after providing all participants with a sufficient explanation of the study and obtaining written informed consent. This study was reviewed by the Wonkwang University Bioethics Committee (WKIRB‐202207‐HR‐059). The general characteristics of the participants, including age, time since stroke, lesion type, paretic side, Brunnstrom recovery stage, and MMSE‐K scores, are summarized in Table 1.

**Table 1:** General characteristics of participants.

Characteristics	Participant A	Participant B	Participant C
Gender/age	Male/72	Male/44	Male/52
Time since stroke (months)	6	15	7
Type of lesion	Infarction	Infarction	Hemorrhage
Paretic side	Right	Right	Left
Brunnstrom recovery stage	Stage 4	Stage 4	Stage 3
MMSE‐K	28	28	29

### 2.2. Design

This study applied the ABA design to a single‐subject experimental research to explain intervention effects. The participants performed 24 sessions over 6 weeks. The baseline period (A) was conducted for four sessions, the intervention period (B) for 16 sessions, and the re‐baseline period (A ^′^) for four sessions.

The baseline period (A) was a period in which only general rehabilitation treatment was applied and was conducted four times in total, with no intervention. The Timed Up and Go (TUG) test and the Box and Block Test (BBT) were used to examine changes in balance, walking ability, and hand dexterity, whereas the Canadian Occupational Performance Measure (COPM), modified Barthel index(MBI), and Fugl‐Meyer assessment (FMA) were used to examine changes in occupational performance, activities of daily living, and upper limb motor function before and after the intervention. To capture quick and immediate treatment effects, only the TUG and BBT, which are simple and require minimal time, were measured repeatedly, whereas the other assessments were conducted only before and after the intervention.

The intervention period (B) was the period during which the general rehabilitation treatment was applied. The telerehabilitation‐delivered LSVT‐BIG intervention was implemented for 16 times in total, 60 min per session, 4 days per week, over 4 weeks. The standardized protocol was implemented (Table 2).

**Table 2:** LSVT‐BIG intervention.

Component	Description
Maximal daily exercises	1. Floor to ceiling (eight rep.)‐seated2. Side to side (eight rep.)‐seated3. Forward step and reach (eight rep.)‐standing4. Sideways step and reach (eight rep.)‐standing5. Backward step and reach (eight rep.)‐standing6. Forward rock and reach (10 rep.)‐standing7. Sideways rock and reach (10 rep.)‐standing
Functional component tasks	Five functional component tasks (five rep)
Hierarchy tasks	1–3 select and are tailored to each individual
LSVT‐BIG walking	Various distances, time

During the re‐baseline period (A ^′^), four repeated measurements were conducted in the same manner as in the baseline period, and occupational performance, daily living activities performance, and upper limb motor function were measured in the last session.

### 2.3. Intervention

The telerehabilitation‐delivered LSVT‐BIG intervention consisted of 16 1‐hour sessions conducted over 4 weeks. Exercises 1 and 2 were performed in a seated position, whereas Exercises 3–7 were performed in sitting or standing depending on the participant′s strength and fall risk. Exercises 1–5 were repeated eight times, and Exercises 6 and 7 were repeated 10 times. Participants maintained an exertion level of at least 7/10, with intensity adjusted by increasing repetitions or changing posture. Short rest periods were provided when participants requested a break. Functional component tasks included one to four daily activities, such as sit‐to‐stand or basic self‐care, each performed five times according to the goals identified using the COPM. Hierarchical tasks involved one to three progressively complex, multistep activities practiced over the 4‐week period. Walking tasks were completed under varying distances, surfaces, and durations to promote maximal movement amplitude during gait.

The telerehabilitation sessions were delivered using the ZOOM videoconferencing platform. To ensure participant safety, an on‐site occupational therapist remained present and supervised all sessions. The intervention took place in a dedicated room specifically arranged for remote rehabilitation, equipped with a high‐resolution camera, a large monitor positioned at eye level, and external speakers to allow clear real‐time audiovisual communication between the participant and the remote therapist. The camera angle was adjusted to capture the participant′s full body during the exercises, enabling the therapist to observe posture, amplitude, and compensatory movements.

Participants performed the exercises by following the therapist′s visual demonstrations and verbal cues displayed on the screen. When support was required, for example, for stability or correction of posture, the on‐site therapist provided appropriate assistance. This setup ensured that the remote therapist could deliver continuous feedback while maintaining participant safety and treatment fidelity.

### 2.4. Instruments

1. TUG test

The TUG test can be used to quickly measure balance and mobility. It measures the time it takes for a participant to stand up from a chair with armrests at the signal “start,” walk 3 m, turn an obstacle, return to the chair, and sit down again. The time is recorded in seconds, and the average of the measured values is calculated from three consecutive measurements. An average of 10 s or less indicates a normal adult, 11–20 s indicates a person with a walking disorder or a frail elderly person, and 30 s or more indicates a high risk of falling requiring an assistive device for walking. The interrater reliability of this scale is 0.98, and the intrarater reliability is 0.99 [24].

2. BBT

The BBT is used to assess upper limb dexterity and hand coordination. This measure consists of a rectangular box measuring 53.7 × 8.5 × 27.4 cm with a center divider and a 2.54‐cm^3^ block of wood. The test involves picking up the block of wood using the affected hand and moving it to the other side as many times as possible for 1 min, and measuring the number of blocks moved. The test–retest reliability of this tool is 0.97 and 0.93 for the right and left hands, respectively [25].

3. FMA

The FMA is a measurement tool designed to evaluate motor function, sensory joint range of motion, balance, and pain level in patients with hemiplegia due to stroke. It consists of 50 items divided into the upper and lower limb areas, and the scores are used individually. The upper limb area used in this study consists of 33 items, including 18 items each for the shoulder, elbow, and forearm; five items for the wrist; seven items for the hand (fingers); and three items for the upper limb coordination ability. Each item is tested thrice on the paretic and nonparetic sides, and the highest score is adopted. The total score is 0–100 points, and 0–2 points are given according to the degree of performance, with 0 points for not performing, 1 point for partial performance, and 2 points for full performance. The interrater and intrarater reliability of this scale is 0.94 and 0.00, respectively, showing high reliability, and the interrater reliability for the upper extremity area is 0.96 [26, 27].

4. MBI

The MBI was modified and supplemented by Shah, Vanclay, and Cooper [28] to evaluate activities of daily living in patients with chronic diseases. It consists of 10 items, including seven self‐care and three mobility ability items. Each item is scored on a 5‐level scale: 0–24 points (complete dependence), 25–49 points (moderate dependence), 50–74 points (moderate dependence), 75–90 points (mild dependence), and 91–99 points (minimal dependence). The total score was 100, indicating complete independence. A higher score can be interpreted as a more independent performance in activities of daily living. This scale has high reliability with an intrarater reliability of 0.89 and an interrater reliability of 0.95 [29].

5. COPM

The COPM is an assessment tool designed by occupational therapists to measure the occupational performance. Through a four‐stage semistructured interview, clients determine their occupational performance in order of priority among tasks that they usually want to do, need to do, and are expected to do in the areas of self‐management, productive activities, and leisure activities, and evaluate their perception of performance and satisfaction using a 10‐point scale (10 = *performs very well*, very satisfied; 1 = *does not perform at all*, not satisfied at all). The score ranges from 1 to 10, with higher scores indicating higher performance and satisfaction.

### 2.5. Procedure

This study conducted interventions in the order of Participants A, B, and C from July 2022 to December 2022. The telerehabilitation‐delivered LSVT‐BIG interventions are shown in Table 2. Standardized: A reference protocol was composed [30]. The telerehabilitation‐delivered LSVT‐BIG training was conducted via a remote system (ZOOM) by a certified occupational therapist. Participants completed the intervention in an independent space under the real‐time supervision of the occupational therapist to ensure the curriculum was followed accurately. The intervention was conducted four times a week lasting 1 h each for 4 weeks at the same time (2:00–3:00 PM) for a total of 16 sessions. The order of the intervention was as follows. First, maximal daily exercises consisting of seven movements were performed. Second, walking was performed by adjusting the distance, time, and surface that the participants could perform using large movements. Third, the functional components and hierarchical tasks selected by the participants were trained.

### 2.6. Statistical Analysis

In this study, the TUG and BBT measurements collected through repeated measurements for each session were analyzed using a visual analysis method with graphs. In order to verify the significance of the measurement values, it was interpreted that there was a significant change when the values among the measurements of the intervention period were higher than the standard ±2 standard deviations of the Baseline A period for two or more consecutive sessions [31]. The FMA, COPM, and MBI measurements collected before and after the intervention were presented as average values to compare the amount of change.

## 3. Results

### 3.1. Results of TUG Test

The TUG test results for each participant, showing changes across the baseline phase (A), intervention phase (B), and re‐baseline phase (A ^′^), are shown in Figure 1. The average score changes for each phase are shown in Table 3.

Figure 1Change in Timed Up and Go.

**Figure:**
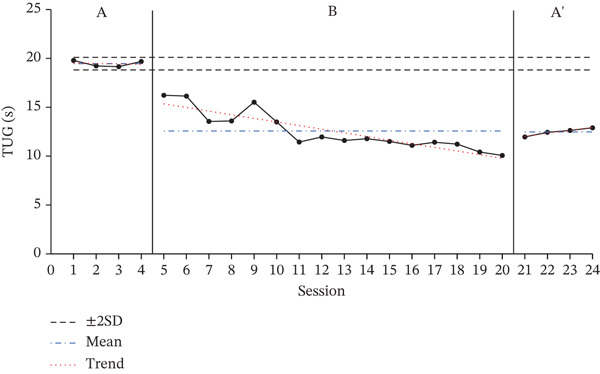
(a)

**Figure:**
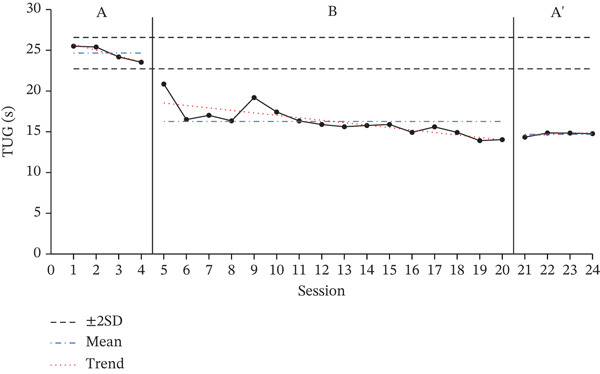
(b)

**Figure:**
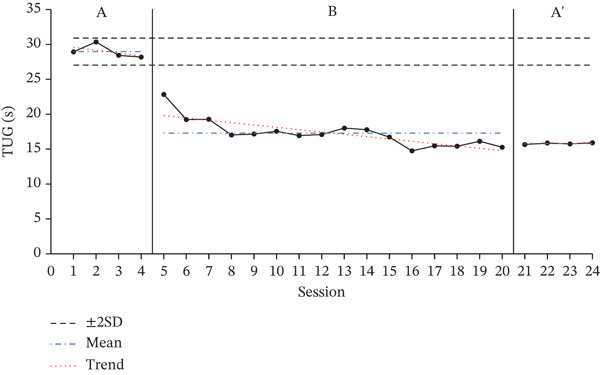
(c)

**Table 3:** Results of the TUG test.

	Baseline A	Intervention B	Baseline A ^′^
	(*M* ± *S* *D*)	(*M* ± *S* *D*)	(*M* ± *S* *D*)
Participant A	19.46 ± 0.32	12.57 ± 1.97	12.48 ± 0.40
Participant B	24.65 ± 0.96	16.26 ± 1.77	14.69 ± 0.25
Participant C	28.97 ± 0.97	17.29 ± 1.97	15.79 ± 0.12

According to the two standard deviation (2SD) analysis of the TUG results, all participants showed 16 consecutive datapoints below the 2SD range, indicating a statistically significant intervention effect. For all participants, the average time spent on the TUG decreased during the intervention phase compared with the baseline phase, indicating a decrease in performance time.

### 3.2. Results of BBT

The results of the BBT, used to assess the dexterity and hand coordination of the participants′ affected upper limbs, are presented in Figure 2 to show the changes during the baseline period (A), intervention period (B), and re‐baseline period (A ^′^). The average score changes for each period are shown in Table 4.

Figure 2Change in Box and Block Test of affected hand.

**Figure:**
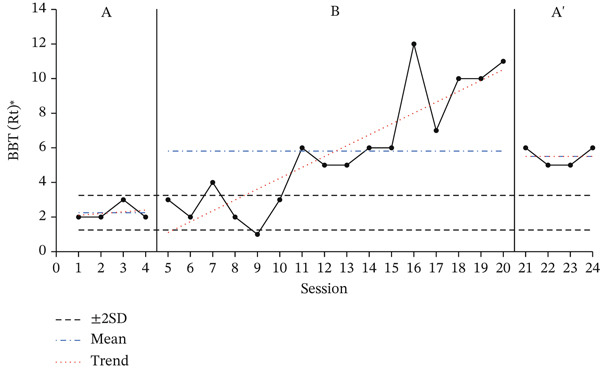
(a)

**Figure:**
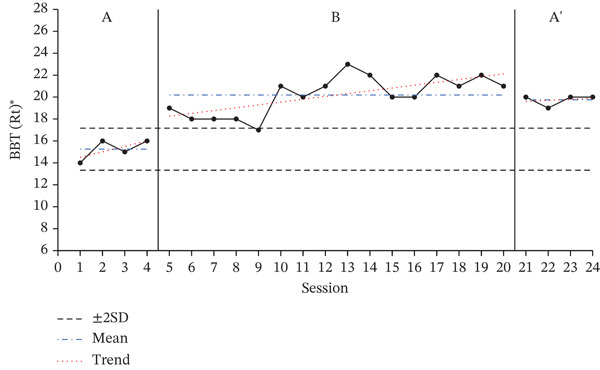
(b)

**Figure:**
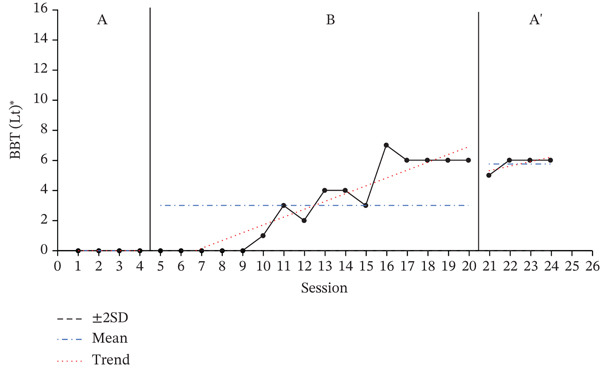
(c)

**Table 4:** Results of the BBT test.

	Paretic side	Baseline A	Intervention B	Baseline A ^′^
		(*M* ± *S* *D*)	(*M* ± *S* *D*)	(*M* ± *S* *D*)
Participant A	Right	2.25 ± 0.50	5.81 ± 3.41	5.50 ± 0.58
Participant B	Right	15.25 ± 0.96	20.19 ± 1.76	19.75 ± 0.50
Participant C	Left	0.00 ± 0.00	3.00 ± 2.63	5.75 ± 0.5

A 2SD analysis of the BBT results showed that all participants had at least two consecutive datapoints above the 2SD range, indicating a statistically significant intervention effect. For all participants, performance improved during the intervention phase, with the average number of BBTs increasing compared to the baseline phase.

### 3.3. Results of Occupational Performance and Daily Living Ability

The performance and satisfaction results for task performance are shown in Table 5. The scores of both performance and satisfaction for all participants increased after the intervention compared with those before the intervention.

**Table 5:** Pre‐ and post‐results of FMA, MBI, and COPM.

	FMA		MBI		COPM			
					Performance		Satisfaction	
	Pre	Post	Pre	Post	Pre	Post	Pre	Post
Participants A	23	26	77	80	3.6	4.8	4.2	5.4
Participants B	29	42	70	80	5	5.8	3.2	4.4
Participants C	20	26	61	66	3.3	4	3.2	4.2

The changes in activities of daily living of participants A, B, and C before and after the intervention are shown in Table 5. The MBI score used to check the changes in activities of daily living showed that all participants′ scores increased after the intervention compared to before the intervention.

## 4. Discussion

This study investigated the effects of the telerehabilitation‐delivered LSVT‐BIG intervention on physical function, activities of daily living, and occupational performance in individuals with chronic stroke. The findings indicate that telerehabilitation‐delivered LSVT‐BIG appears beneficial for improving functional recovery in chronic stroke patients. Across participants, improvements were observed in gait and balance, the upper limb function, activities of daily living, and occupational performance. These results suggest that telerehabilitation‐based delivery of LSVT‐BIG may represent a viable and clinically meaningful alternative to conventional face‐to‐face intervention for individuals with chronic stroke who experience barriers to accessing in‐person rehabilitation services.

LSVT‐BIG was chosen because it applies neuroplasticity principles through standardized, high‐amplitude movements and incorporates functional, hierarchical tasks tailored to individual needs [32]. Telerehabilitation was included in response to increasing needs related to cost, accessibility, and mobility limitations among individuals with stroke, and it has been shown to support continuous therapist supervision and real‐time feedback without geographical constraints [18, 33, 34]. Accordingly, integrating LSVT‐BIG with real‐time telerehabilitation provides an evidence‐informed approach aligned with the practical realities of stroke rehabilitation and supports the clinical relevance of the study design. To examine the changes in physical function following a telerehabilitation‐based LSVT‐BIG intervention in patients with stroke, this study selected validated outcome measures commonly used in previous LSVT‐BIG research. The TUG test was used to assess balance and mobility, as it has been widely applied in studies investigating LSVT‐BIG interventions for individuals with stroke, including those delivered via telerehabilitation [14, 20, 23]. In addition, the upper limb function was evaluated using the BBT, which has been employed in prior LSVT‐BIG studies to measure manual dexterity [9]. The TUG test provides a rapid assessment of functional mobility and balance [24], whereas the BBT offers a simple and reliable measure of gross manual dexterity [35]. In single‐subject research designs, outcome measures must be clearly linked to defined dependent variables and allow for a valid, consistent, and repeated assessment across experimental phases [36]. Accordingly, both the TUG and BBT were considered appropriate for repeated measurement and were therefore selected as outcome measures in this study.

Following the telerehabilitation‐delivered LSVT‐BIG intervention, all participants demonstrated overall improvements in gait and balance as measured by the TUG. These findings are consistent with previous studies reporting improvements in gait and balance after LSVT‐BIG intervention in patients with stroke [14], as well as prior telerehabilitation studies showing positive effects on gait and balance in patients with Parkinson′s disease [20, 23]. The observed improvements may be attributed to a large‐amplitude movement training, a core component of LSVT‐BIG, which is thought to recalibrate individuals′ perception of movement amplitude and accuracy. This recalibration likely contributes to an enhanced functional mobility and postural control, thereby improving gait and balance performance. In addition to lower extremity outcomes, improvements were also observed in the upper limb function. Positive changes in manual dexterity were demonstrated by increased BBT scores, and all participants showed significant improvements in upper extremity motor function as measured by the FMA. These results align with previous studies reporting significant gains in the upper limb function following LSVT‐BIG intervention in patients with stroke [11, 14]. Furthermore, studies applying repetitive amplitude training using LSVT‐BIG in individuals with Parkinson′s disease have reported more accurate arm movements and significant improvements in proprioceptive performance, suggesting a readjustment of proprioceptive processing [37]. In the present study, the LSVT‐BIG intervention incorporated high‐intensity, repetitive movements of both the upper and lower extremities, combined with large trunk movements and functionally hierarchical task activities involving upper limb use. This comprehensive movement approach may explain the observed improvements in upper limb outcomes and supports the clinical applicability of telerehabilitation‐delivered LSVT‐BIG for upper extremity rehabilitation, which has not been sufficiently examined in previous telerehabilitation studies.

All participants demonstrated significant improvements in occupational performance and satisfaction as measured by the COPM, and the MBI results indicated positive changes in activities of daily living. These findings are in line with previous studies reporting improvements in occupational performance, satisfaction, and daily functioning following LSVT‐BIG interventions [11–13]. Prior research suggests that improvements in occupational performance and satisfaction may be related to the inclusion of meaningful and motivating tasks within the LSVT‐BIG protocol [12]. In addition, one‐on‐one therapist–participant interaction, including direct cueing and encouragement, has been identified as a factor that can enhance confidence and engagement in task performance [11]. Functional and hierarchical task training grounded in everyday activities may have contributed to the observed improvements in activities of daily living. Although the intervention was delivered remotely, real‐time one‐on‐one interaction was maintained in a manner comparable with face‐to‐face intervention. Repeated practice of personally meaningful tasks may have enhanced motivation and promoted gains in daily activity performance.

This study provides preliminary evidence supporting the clinical applicability of telerehabilitation‐delivered LSVT‐BIG. Several limitations should be considered when interpreting the findings of this study. First, it was a single‐subject study targeting three patients with stroke, which limits the generalizability of the results. Second, follow‐up studies and re‐evaluations were not conducted to confirm the continued effects of the intervention. Third, differences in telerehabilitation delivery methods and protocols across studies may limit direct comparisons with existing telerehabilitation research. Future research should examine telerehabilitation‐delivered LSVT‐BIG using larger samples and more rigorous study designs, incorporate follow‐up assessments, and further clarify which intervention components are most strongly associated with functional outcomes.

## 5. Conclusion

This study is aimed at investigating the effects of LSVT‐BIG intervention delivered via telerehabilitation on physical function, occupational performance, and activities of daily living in patients with stroke, and to assess its clinical applicability. After applying LSVT‐BIG intervention through telerehabilitation, the physical function of stroke patients significantly improved, and their performance and satisfaction in occupational performance increased. Although the degree of change in activities of daily living varied among participants, significant improvements were observed. Therefore, this study suggests that LSVT‐BIG intervention delivered via telerehabilitation may be utilized as a multifaceted approach for patients with stroke.

## Author Contributions

Conceptualization: S‐A.J. and D‐G.H.; methodology: D‐G.H.; validation: D‐G.H. and I‐T.C.; formal analysis: S‐A.J.; investigation: S‐A.J. and I‐T.C.; writing—original draft preparation: S‐A.J. and I‐T.C.; writing—review and editing: S‐A.J. and D‐G.H.; supervision: D‐G.H.

## Funding

This study was supported byWonkwang University in 2024 (10.13039/501100002569).

## Disclosure

All authors have read and agreed to the published version of the manuscript.

## Conflicts of Interest

The authors declare no conflicts of interest.

## Data Availability

The data supporting the findings of this study are available from the corresponding author upon request; however, they are not publicly disclosed due to privacy and ethical restrictions.
